# Curie temperature engineering in a novel 2D analog of iron ore (hematene) *via* strain†

**DOI:** 10.1039/d0na00556h

**Published:** 2020-11-22

**Authors:** Renu Singla, Timothy A. Hackett, Sarvesh Kumar, Jyotsna Sharma, Manish K. Kashyap

**Affiliations:** Department of Physics, Kurukshetra University Kurukshetra 136119 Haryana India manishdft@gmail.com mkumar@kuk.ac.in +91-1744-238277 +91-1744-238410 extn 2482; Department of Biochemistry, University of Nebraska–Lincoln Lincoln Nebraska 68588-0664 USA; Inter-University Accelerator Centre (IUAC) Aruna Asaf Ali Marg New Delhi 110067 India; Department of Physics, Amity School of Applied Sciences, Amity University Haryana Gurugram 122413 India

## Abstract

As a newly exfoliated magnetic 2D material from hematite, hematene is the most far-reaching ultrathin magnetic indirect bandgap semiconductor. We have carried out a detailed structural analysis of hematene *via* prefacing strain by means of first-principles calculations based on density functional theory (DFT). Hematene in the pristine form emerges out to be a magnetic semiconductor with a bandgap of 1.0/2.0 eV for the majority/minority spin channel. The dependence of magnetic anisotropy energy (MAE), *T*_C_, and the bandgap on compressive and tensile strains has been scanned exclusively. It is examined that *T*_C_ depends firmly on the compressive strain and increases up to 21.1% at a compressive strain of 6% whereas it decreases significantly for tensile strain. The MAE is negatively correlated with the tensile and compressive strain. The value of MAE for all compressive strain cases is more than that of the pristine hematene. These results summarize that the studied 2D hematene has broad application prospects in spintronics, memory-based devices, and valleytronics.

## Abstract 2

Hematene: 3 atoms thick 2D material with high *T*_C_.

## Graphical abstract

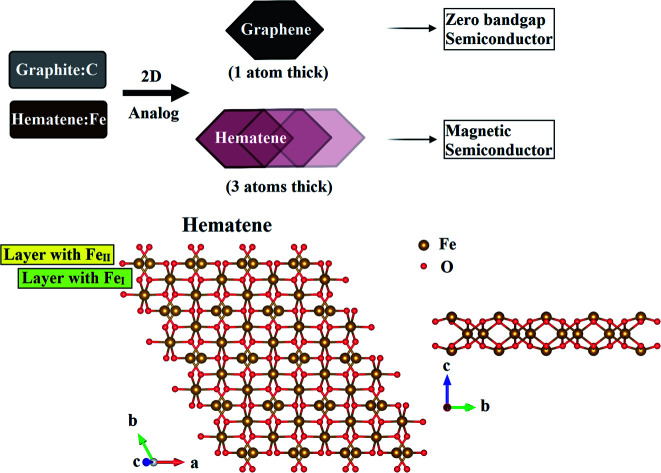

## 1 Introduction

Research interests in two-dimensional (2D) materials have rapidly been increasing over the past two decades.^1,2^ Graphene is the first 2D material that was isolated from graphite by using the scotch tape technique in 2004 at The University of Manchester.^1^ It is 200 times stronger than steel, lightweight, flexible, and more conductive than copper. Its unique superlative physical and chemical properties make it a promising material for various electronic and photonic applications.^3–5^ Since 2004, many experimental and theoretical studies have been performed over graphene-based materials and they have been proved masters in almost every field especially in catalysis, electronics, photovoltaics, electrochemistry, and optoelectronics.^6,7^ As a result of these tremendous applications, material scientists have started to explore the worth of 2D materials for magnetism as well. According to the Mermin–Wagner theorem,^8^ long-range ferromagnetic order present in a bulk material can easily be destroyed by the thermal fluctuations for dimensions, *d* ≤ 2. The animation of an aligned arrangement of magnetic moments over macroscopic length scales is the trademark of magnetism with an intuitive disturbing of time-reversal uniformity. This is frequently determined by the interplay of the adjacent spins (exchange interactions) that leads to accommodate only some specific correlative orientations among all. At zero temperature, this local order can extend over macroscopic length scales. With a rise in temperature, thermal fluctuations tend to obstruct the alignment of magnetic moments in the neighboring regions. In this way, long-range order is destroyed above the Curie temperature (*T*_C_). This is the main parameter that decides whether the magnet can be used in numerous theoretical, experimental, and technological advances such as the study of topology, the fluctuation driven in new phases, electronic manipulation, and detection of spin.^9–12^ Therefore, *T*_C_ must be at least of the order of room temperature to serve as a new platform for various technological applications. These facts disheartened material scientists; however, they have not completely given up over this area.

The other members of the family of next-generation 2D materials belong to the monolayers of CrI_3_ and CrGeT_3_ which have also been fabricated successfully.^13,14^ After that, researchers regained their passion for 2D magnetism and hematene is the latest member in this category which is 2D analog of hematite ore. Hematene has several properties in common with graphene. As graphene is a thin sheet of carbon atoms, hematene is also a thin sheet of iron and oxygen atoms. Researchers at Rice University synthesized 2D hematene with the liquid-phase exfoliation technique.^15^ Balan *et al.*^15^ confirmed the 2D morphology of hematene with the help of transmission electron microscopy. They reported the existence of long-range ferromagnetic order in hematene while its parent hematite is antiferromagnetic in nature. They also showed that hematene on loading with arrays of titanium nanotubes may serve as an enhanced excellent photocatalyst. The atoms in hematene are found to be held together by strong chemical bonds rather than weak van der Waals interactions. Therefore, hematene has the potential to serve as a replacement for well-known 2D materials such as graphene, black phosphorous, and MoS_2_ in various applications where the structures of these eminent materials can be easily disrupted. This fact revolutionized the scenario of emerging 2D magnetic materials.

Bandyopadhyay *et al.*^16^ investigated the nature of magnetic ordering in this 2D metal oxide hematene. They explained that it has a stripped ferrimagnetic ground state with a feeble overall magnetic moment. They also attempted to modulate its magnetism with alloying and by substituting chromene (Cr_2_O_3_) instead of Fe_2_O_3_. Gonzalez *et al.*^17^ studied the structural, optical and electronic properties of hematene. Further, they also explored the functionalization of hematene with Au (111) and stanene (a hexagonal lattice with its size matching with that of the hexagonal sublattice of hematene). The interaction with the former is weak, involving a long bonding distance, just like a typical van der Waals system whereas the interaction with the latter leads to the hybridization of the p orbital of stanene with the unoccupied d_*z*^2^_ orbitals of hematene, turning the resultant heterostructure into a ferrimagnet. Chen *et al.*^18^ discussed tuning the magnetism of two-dimensional hematene by ferroelectric polarization and found that the control of magnetism depends on the interface terminations. This occurs mainly due to change in the Fe–O bond length, which is determined by relative displacement of Fe and O atoms and electron transfer between five nonequivalent 3d orbitals.

The search for high *T*_C_ in 2D materials presents a major challenge. In these critical situations, hematene may be the only 2D material having a *T*_C_ of the order of room temperature with a remarkable breakthrough for advanced magnetic applications. In the present study, we focus on the novelty of predicting the *T*_C_ of pristine hematene first by *ab initio* simulations. Further, the 2D materials crystals can sustain large strains compared to their bulk counterparts. For example, single-layer FeSe can sustain strains as large as 6%, whereas single-layer MoS_2_ can be strained as high as 11%.^19–21^ Therefore, strain engineering can also be a practical approach to tune the properties of 2D materials. The second aim of our study is to check the effect of compressive and tensile strains up to 6% on the magnetic response and *T*_C_. To our firm belief, this is the first report on the prediction of *T*_C_ of hematene and its strain-induced magnetism.

## 2 Calculation methodology

The *ab initio* calculations were carried out by means of the projector augmented wave (PAW) method based on density functional theory (DFT) as implemented in the Vienna *ab initio* Simulation Package (VASP).^22,23^ The coulomb corrected generalized gradient approximation (GGA + U) within Perdew–Burke–Ernzerhof (PBE) parameterization was employed in order to take into account exchange-correlation potentials.^24^ The Hubbard parameter, *U* = 4 eV was used for correcting onsite Fe-d electron correlation in hematene.^25^ Further, the value of the U-parameter was also optimized and *U* = 4 eV was selected because it reproduces the experimental bandgap of pristine hematene quite well (details are presented in ESI Fig. S1 and Table S1†). A vacuum layer of 15 Å thickness was added along the *c*-axis to avoid periodic interactions. This choice was made after performing the convergence test by varying the vacuum distance from 12 to 20 Å, as shown in ESI Fig. S2.† To mimic the 2D lattice, the energy cut off for the plane waves was set to 520 eV and the Brillouin zone was sampled by using 15 × 15 × 1 mesh of *k*-points. Very tight energy convergence criteria of 10^−8^ eV were used for electronic relaxation. We performed a conjugate-gradient algorithm to relax the ions into their ground state until the Hellmann–Feynman force on each atom became less than 0.001 eV Å^−1^. During the relaxation, the Gaussian method with smearing width = 0.2 eV was utilized to compute partial occupancies for each orbital whereas the electronic density of states (DOS) was calculated by using Blöchl correction in the tetrahedron method with the same smearing width. The thermal stability of hematene at a temperature of 300 K was checked in VASP by employing the NVT ensemble.^26^ The phonon spectrum was obtained by utilizing the Density Functional Perturbation Theory (DFPT) approach and the post data were processed with the phonopy code.^27^ Bader charge analysis was executed to study the charge transfer and charge redistribution.^28^ The value of *T*_C_ was estimated within mean-field theory by computing various exchange constants that are related to the magnetic interactions between the Fe atoms of the same and different sublattices with the help of Heisenberg Hamiltonian. In order to predict MAE, non-collinear calculations were carried out with the inclusion of spin–orbit coupling (SOC) non-self consistently by changing the direction of the quantization axis.

## 3 Results and discussion

### 3.1 Pristine hematene

**Fig. 1:**
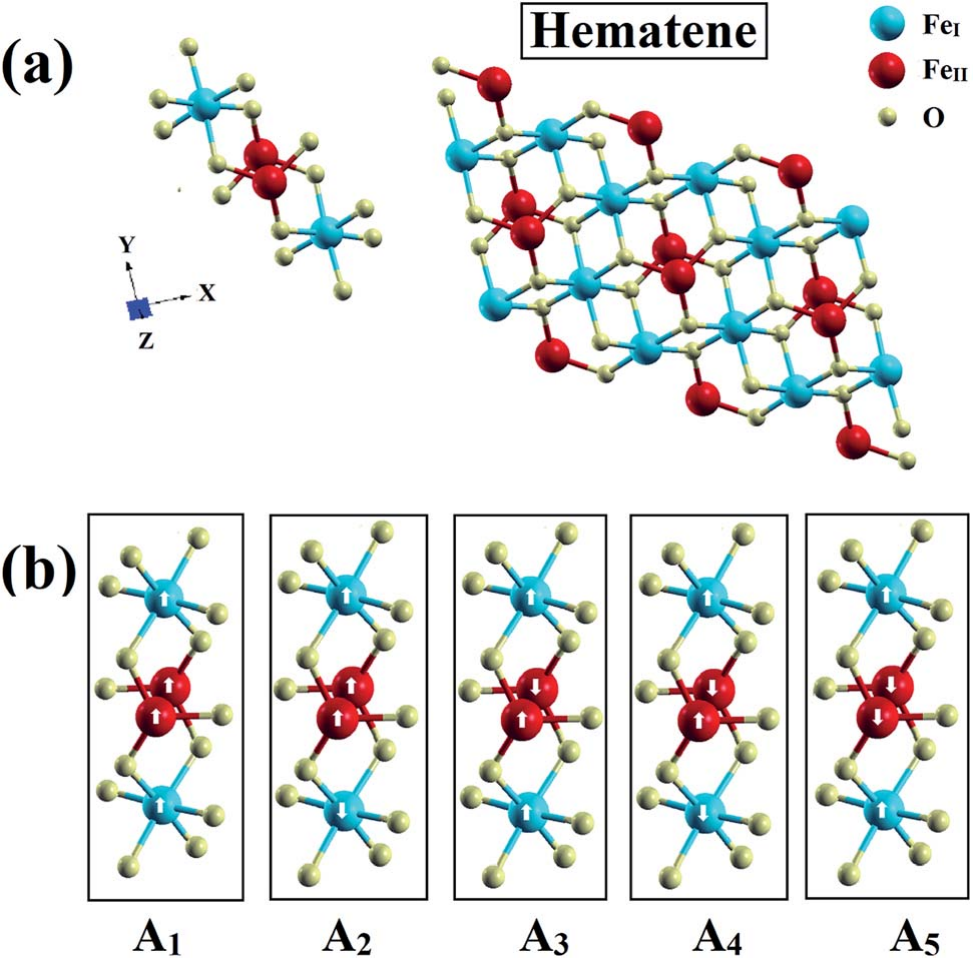
(a) Schematics of the hematene monolayer with three atoms and (b) visualization of its various possible magnetic configurations.

The novel 2D material hematene is not a single atom thick sheet like monolayer graphene; however, it has a thickness of three atoms only (two inequivalent Fe atoms and one O atom). But, this can still be categorized as a monolayer. The first Fe atom (Fe_I_) is enclosed in an octahedron of six O-atoms whereas the other one (Fe_II_) is surrounded by three O-atoms as depicted in Fig. 1(a). It was exfoliated from hematite experimentally in two phases along the [001] and [100] directions;^15^ however, hematene (001) possesses exciting lattice symmetries and is found to be more stable. Therefore, we considered mainly this phase. It has a triangular lattice with a lattice constant, *a* = 5.09 Å.^17^

**Fig. 2:**
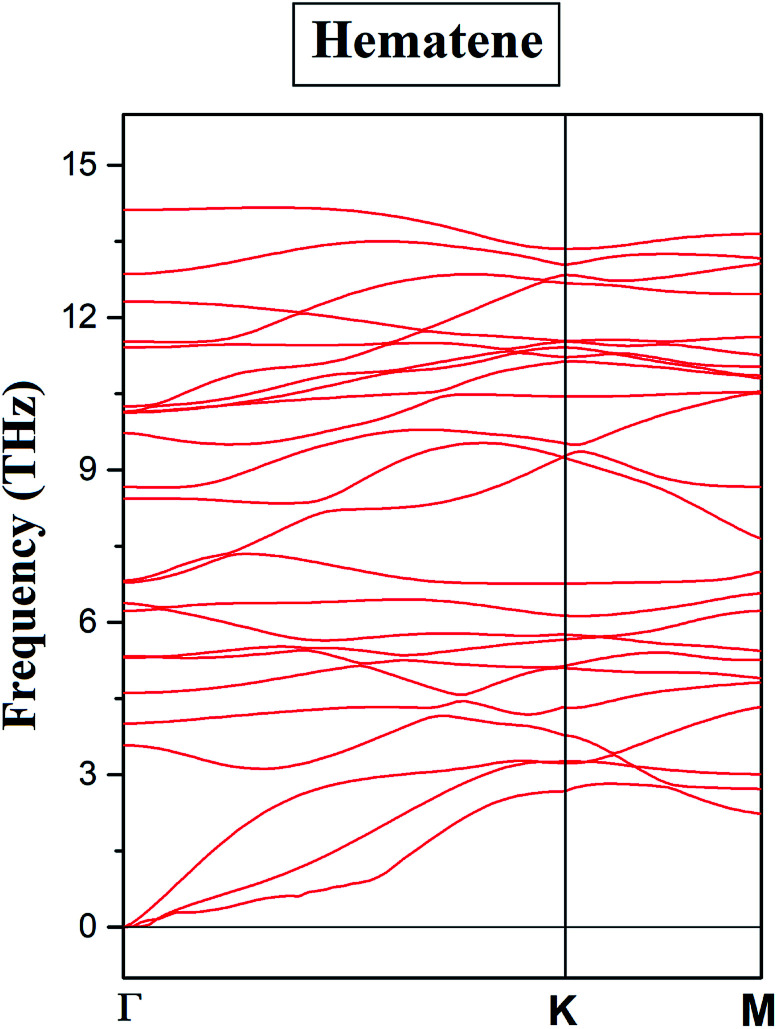
Phonon dispersion curve for hematene in A_5_ configuration along the high symmetry *k*-points.

**Fig. 3:**
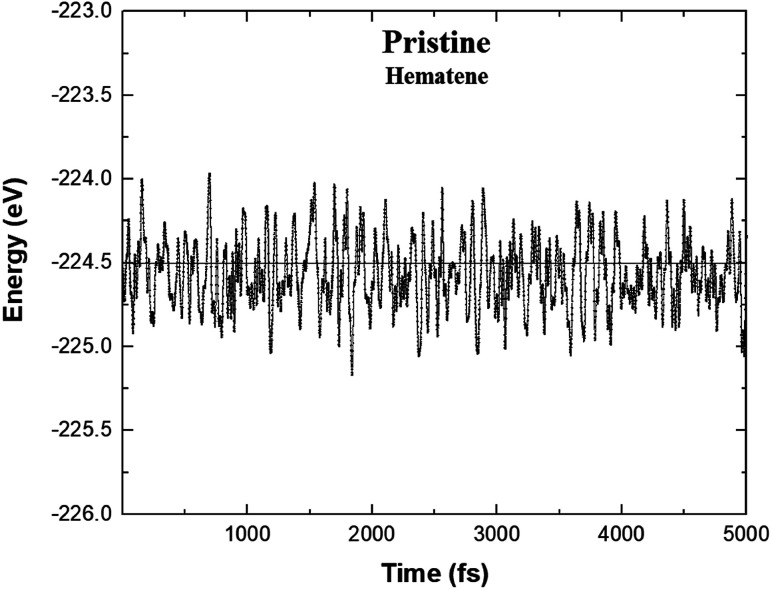
Variation of the total energy of hematene in A_5_ configuration during AIMD simulations at 300 K.

Based on the environment of the two Fe atoms, various magnetic configurations can be designed. In fact, both inequivalent Fe atoms, Fe_I_ and Fe_II_, have an occupancy of two in the monolayer (*i.e.* two Fe atoms each in two different sublattices) and thus, to consider ferromagnetic and antiferromagnetic states, five configurations A_1_, A_2_, A_3_, A_4_ and A_5_ as depicted in Fig. 1(b) and also in Table 1, can be possible. To check the structural stability of these configurations, the formation energies (*E*_for_) were evaluated by using the following expression:

1 *E*_For_ = *E*_Hema_ − 4*E*_Fe_ − 6*E*_O_

where *E*_Hema_, *E*_Fe_ and *E*_O_ are the ground state energies of hematene in a particular magnetic configuration, Fe and O atoms, respectively. The negative value of formation energies for all cases, as listed in Table 1, confirm their stability. Out of the five magnetic configurations, A_5_ (for which two Fe_I_ atoms in one sublattice align antiparallel to two Fe_II_ atoms in the second sublattice) is found to be most stable. To ensure the dynamical stability of this configuration, phonon dispersion spectrum was analyzed (Fig. 2), which reflects the real phonon frequencies. Therefore, hematene in A_5_ configuration is dynamically stable even in the absence of a substrate. Further, we determined the thermal stability of this configuration at room temperature (300 K) with a time step of 1 fs by using *ab initio* Molecular Dynamics (AIMD) simulations.^26^ After running 5000 steps, the movements of Fe and O atoms were found to be just minor with negligible changes in the bond length. Also, there was no trace of breaking of bonds, and the variation in total energy is also very small (Fig. 3) which indicates that hematene can handle thermal fluctuations very well.

**Table 1:** Calculated formation energy and total spin magnetic moment (*μ*_stot_) of hematene in different magnetic configurations. ↑/↓ represents majority/minority spin electrons

State	Fe_I_	Fe_II_	*E*_For_ (eV)	*μ*_stot_ (*μ*_B_)	Nature
A_1_	↑↑	↑↑	−0.430	16.10	Ferromagnetic
A_2_	↑↑	↑↓	−1.175	8.31	Ferromagnetic
A_3_	↑↓	↑↑	−1.198	7.80	Ferromagnetic
A_4_	↑↓	↑↓	−1.432	0.00	Antiferromagnetic
A_5_	↑↑	↓↓	−1.639	0.52	Ferrimagnetic

The magnetic response of hematene as governed by DFT simulations under GGA + U indicates the spin magnetic moment (*μ*_s_) of Fe_I_ as 3.896 *μ*_B_ and Fe_II_ as 4.156 *μ*_B_, aligning in antiparallel arrangement with the total spin magnetic moment (*μ*_stot_) as 0.52 *μ*_B_. This confirms its ferrimagnetic nature which is in accordance with the previous results by Balan *et al.*,^15^ Bandyopadhyay *et al.*^16^ and Gonzalez *et al.*^17^ The magnetic interaction mechanism is quite compatible with the kinetic exchange system in this case. Although the Fe_II_–Fe_II_ distance is large, yet Coulomb repulsions decrease rapidly like hoping strength. Also, Fe_I_–Fe_I_ and Fe_II_–Fe_II_ interactions are intervened by the O-atom leading to magnetic superexchange as the bond angle of Fe_I_–O–Fe_I_ is ∼90°. But the O-atom here is threefold coordinated and forms sp^2^ bonding, halting its strong FM nature. This result is in accordance with the experimental hysteresis data^17^ which shows that when the external field is removed, hematene has a total spin moment of 4 *μ*_B_ which is about 1/4 of that of A_1_ state. So, this experimental remanence and feeble net magnetic moment in A_5_ are due to the difference in the charge distribution of Fe atoms from both sublattices which is explained later on in electronic properties. On the contrary, bulk hematite is antiferromagnetic in nature and its Morin transition occurs at 260 K with a Neel temperature of 955 K. Being the most stable configuration, we have considered A_5_ configuration only for detailed analysis of electronic properties and the magnetic response.

**Fig. 4:**
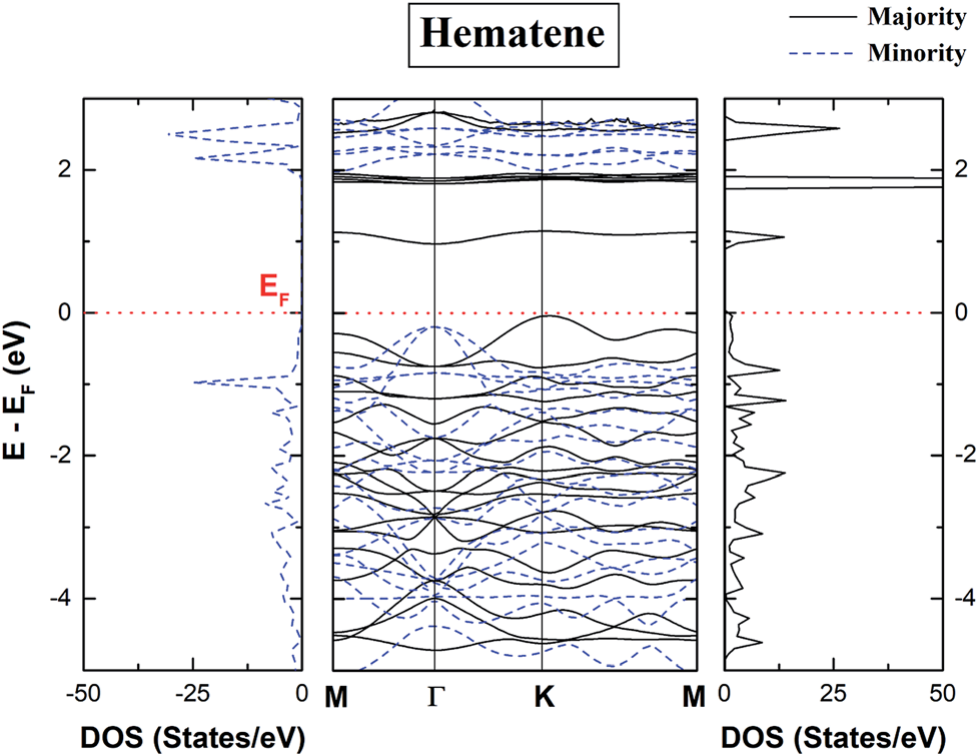
Calculated spin polarized total density of states (TDOS) and bandstructure of pristine hematene. The solid/dotted line shows contributions from the majority/minority soin state. Here, *E*_F_ is shifted to 0 eV.

The spin-polarized total density of states (DOS) and bandstructure of pristine hematene (Fig. 4) indicate the generic DOS in nature and different band gaps for both spin channels which is a general property of a magnetic semiconductor. A bandgap of ∼1.0/1.9 eV in the majority/minority spin channel is obtained; on checking the bandstructure, this gap is found to be indirect along *Γ*–*K* for the majority/minority spin channel.

**Fig. 5:**
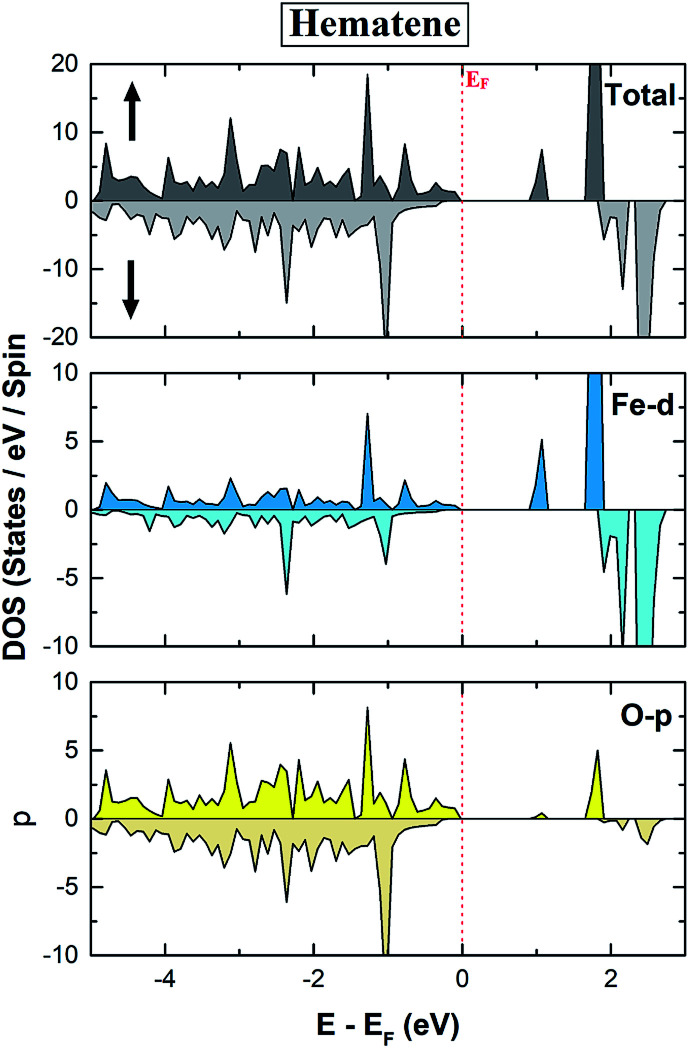
Calculated total and orbital resolved spin polarized DOS of pristine hematene.

The electronic properties of hematene are quite interesting especially in the vicinity of the Fermi level (*E*_F_). The main contribution in the total DOS comes only from Fe-d and O-p states (Fig. 5). The lowest conduction band is highly localized, has mainly the contribution from Fe-d orbitals, and hardly forms Blöch orbitals whereas the highest valance bands are delocalized due to hybridization between Fe-d and O-p states. The splitting in the majority and minority spin channels is evident from the fact that it has ferromagnetic order within each sublattice and antiferromagnetic order within the whole lattice.

**Fig. 6:**
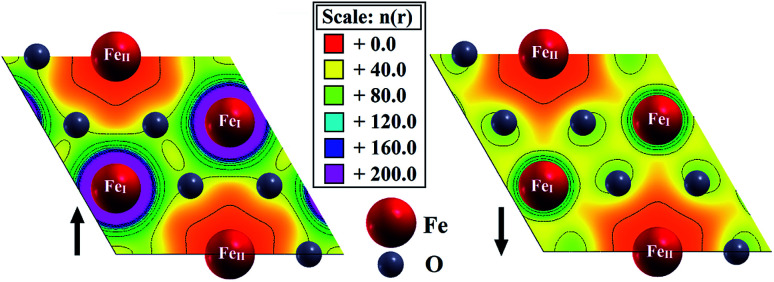
Spin resolved electronic valance charge density of pristine hematene.

The valence charge density distribution (Fig. 6) of pristine hematene is different in both spin channels since each Fe_I_ has three Fe_II_ atoms as the first nearest neighbors and each Fe_II_ has six Fe_I_ atoms as the first nearest neighbors. This implies that the Fe_I_–Fe_II_ interactions are more prominent. Also, Fe_I_ has a spherical electron cloud whereas, for Fe_II_, the electron cloud is delocalized differently in different directions. Also, the difference in the charge distribution for both spins is confirmed further by Bader charge analysis (Table 2). Each Fe_I_ atom donates 1.74*e* to the O-atom whereas each Fe_II_ atom donates 1.55*e* to the same.

**Table 2:** Average charges (in electrons) for hematene calculated by Bader charge analysis

Atom	Average charges
Fe_I_	+1.74*e*
Fe_II_	+1.55*e*
O	−0.94*e*

The MAE originates due to the dependence of the magnetic properties of a material on a particular direction. Classically, it is due to dipole–dipole interactions but quantum mechanically, its origin is in SOC. The consequence of SOC includes anisotropic exchanges, both the symmetric and antisymmetric Dzyaloshinskii–Moriya Interaction (DMI), and magnetocrystalline anisotropy (MCA). As hematene has inversion symmetry, so, no DMI is expected in its pristine form. The magnetism in 2D materials has been examined extensively for decades and serves as a testbed for their study in different aspects such as critical behavior and dimensional crossover of magnetic ordering. The interplay of MCA and dimensionality is considered to be a promising route for the development of magnetism in 2D materials as it is only MCA that can stop thermal fluctuations to destroy long-range magnetic order. In this regard, first principles DFT calculations that entail SOC authorize a dominant theoretical tool to compute MCA. Howbeit, the tiny magnitude of the associated MAE enforces stringent convergence to the calculations at the rate of high computational cost. This purpose was realized by first characterizing the Hamiltonian of the system including the scalar relativistic term self-consistently and then including the SOC non-self-consistently within the force theorem^29–31^ by changing the direction of the quantization axis. The SOC Hamiltonian is predominantly expressed as a sum of one-electron operators:2
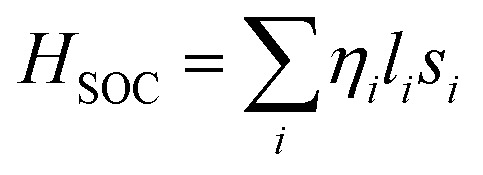
Here *l*_*i*_ and *s*_*i*_ account for the orbital and spin momentum operators of the *i*^th^ electron, respectively, and *η*_*i*_ stands for the SOC strength. The MAE is finally calculated as follows:^32^

3 MAE = Δ*E*_tot_^*x*^ − Δ*E*_tot_^*z*^ ≅ *E*_band_^*x*^ − *E*_band_^*z*^

where Δ*E*_tot_^*x*/*z*^ represents the total energy change along the *x*/*z* direction and *E*_band_^*x*/*z*^ is the band energy in the vicinity of *E*_F_ along the *x*/*z* direction. The positive/negative value of MAE indicates uniaxial/in-plane magnetic anisotropy. The MAE for pristine hematene is found to be uniaxial with a value of 87.05 μeV per Fe atom. Since, the value of MAE is very small with μeV precision, the dependence of its value on energy cut off was also cross-checked (ESI Fig. S3†).

### 3.2 Strained induced effects in hematene

After considerable analysis of the structural, electronic, and magnetic response of pristine hematene, the robustness of the magnetic response was checked under the effect of the strain. In this context, two types of volumetric strains *i.e.* compressive and tensile strain (in the range: −6% to +6%) were considered. The relaxations of strain-induced structures do not induce any distortion in the shape of hematene. The stability of these strained structures was confirmed with the help of formation energy and the phonon spectrum. The negative values of formation energies and real phonon frequencies for all cases illustrate their stability (ESI Fig. S4 and S5,† respectively).

**Fig. 7:**
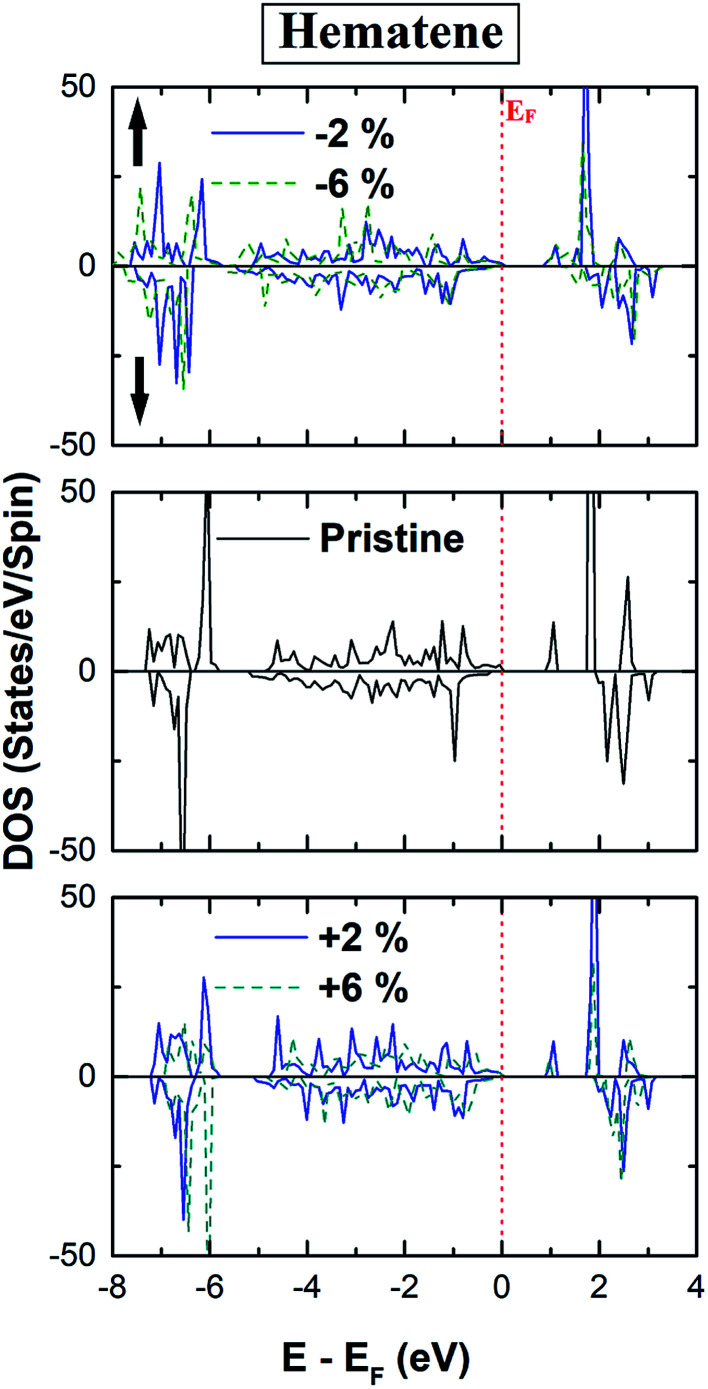
Calculated spin polarized total density of states (TDOS) of hematene in the strain range (−6% to +6%).

The total DOS in the vicinity of *E*_F_ remains unaffected for all cases as depicted in Fig. 7. Further, there is hardly any change in the bandgap for both spin channels, implying that this gap is robust under the applied range of strain. However, we have noticed significant changes in the magnetic response such as magnetic moments, MAE, and *T*_C_. As strain modifies the lattice parameters, there will be a concomitant adaptation of band dispersion. In itinerant materials, this may lead to an appreciable change in *μ*_stot_ if the ground state is modified due to the effect of strain. But here ground state configuration remains intact with strain and a diminutive change in *μ*_stot_ is observed for all cases. Despite this, *μ*_stot_ increases slowly with the compressive strain and decreases a bit with the tensile strain (Table 3). In broad terms, this fragile escalation is a result of the fact that with the increase of compressive strain, the number of electrons forming the covalent bond decreases, which yields more electrons located at the Fe atom. Also with the increase in compressive strain, the majority and minority spin channels differ more in energy, respectively, enhancing the net residual *μ*_stot_.

**Table 3:** Strain-induced magnetic response, Curie temperature (*T*_C_) and easy axis of hematene

Strain	*μ*_stot_ (*μ*_B_)	MAE (μeV per Fe atom)	*J* (meV)	*T*_C_ (K)	Easy axis
−6%	0.562	87.85	20.41	357	Out of plane
−4%	0.546	88.81	19.29	337	Out of plane
−2%	0.534	90.43	18.15	317	Out of plane
0%	0.520	87.05	16.80	295	Out of plane
2%	0.518	86.09	15.70	274	Out of plane
4%	0.512	81.50	14.58	254	Out of plane
6%	0.502	74.70	13.45	234	Out of plane

The symmetry of hematene is hexagonal, so MAE at first order, for a given polar angle (*θ*) is given by:4
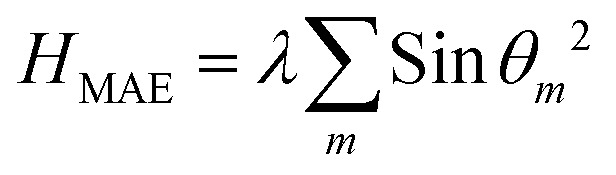
where *m* runs over each Fe atom, *θ*_*m*_ is the angle between the *m*^th^ Fe atom and normal to the symmetry plane. *λ* is the quadratic contribution to the energy. We have also examined in which manner, MAE changes with the biaxial strain (Table 3). The magnitude of MAE per Fe atom decreases/increases for an increase in tensile/compressive strains and has a maximum value of 90.43 μeV at −2% strain. The easy axis remains off the plane for all cases.

The various exchange constants (*J*'s) that are related to the magnetic interactions between Fe atoms of the same and different sublattices are computed using Heisenberg Hamiltonian within the mean field theory^33^ as follows:

5 *E*_FM_ − *E*_AFM_ = 8*JS*^2^

**Fig. 8:**
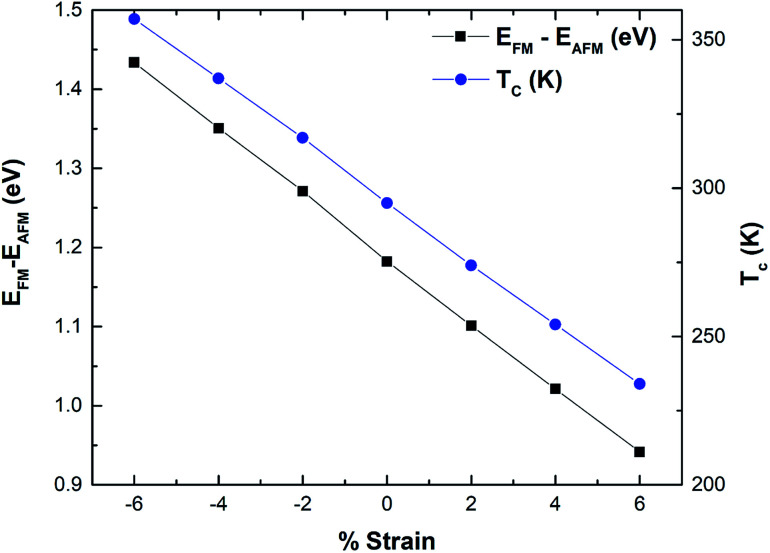
Variation of the energy difference mainly between the FM and AFM phase and *T*_C_ as a function of strain for hematene.

The magnetism in hematene mainly arises from the partially filled d orbitals of Fe atoms as the valence electronic configuration of the Fe atom is 3d^6^4s^2^ and the O atom has an electronegativity of 2. Since, there are six O and four Fe atoms per unit cell, Fe exists in the +3 state and the valance state becomes 3d^5^. This means here |*S*| = 5/2. We can easily calculate the energy difference between FM and AFM states using the DFT approach and estimate *J* by using eqn (5). This difference is negatively correlated with strain (Fig. 8). After that, the *T*_C_ of hematene can be computed by using the expression:^34^6
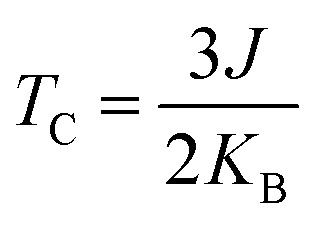


The calculated *T*_C_ for pristine hematene is found to be 295 K which is quite close to room temperature. We made a further attempt to tune it above room temperature. Table 3 clearly shows that it is highly sensitive to both types of strains. *T*_C_ increases well above room temperature with the compressive strains and decreases with the tensile strain (Fig. 8). The ultimate increase in *T*_C_ is of 21.1% on compacting the lattice parameter by 6%. This may be due to the fact that exchange interactions are of short-range and increase with a decrease in distance. Through effective compressed strain engineering, all the electronic properties of hematene remain intact with a feeble change in *μ*_stot_ and MAE, but we achieved appreciable improvement in *T*_C_ (far above room temperature). This opens up new avenues for hematene to serve in spintronic and memory-based magnetic devices.

## 4 Conclusions

In summary, we have examined 2D-hematene by strain engineering with an aim to upgrade the magnetic response using first-principles calculations based on DFT. Pristine and all strain-induced hematene structures in the ground state are found to be a magnetic and indirect semiconductor with different bandgaps for the majority and minority spin channels. The main drawback of low *T*_C_ which forbids 2D materials from showing their potential towards magnetism related applications has been overcome in hematene because for its pristine form and all strain-induced structures, high *T*_C_ is observed. On applying compressive and tensile strains, the structural and electronic properties do not change much but the magnitude of MAE and *μ*_stot_ increase for all compressive strain cases as compared to the pristine case. We hope that our results will further stimulate experimental studies on hematene and pave the way for its application in magnetic tunneling junctions and magnetic random access memory devices.

## Conflicts of interest

There are no conflicts to declare.

## Supplementary Material

NA-002-D0NA00556H-s001
