# Supplementary material for: Curie temperature engineering in a novel 2D analog of iron ore (hematene) via strain
Source: Nanoscale Adv. 2020 Nov 22;2(12):5890–6. doi: 10.1039/d0na00556h (PMC9417893; doi:10.1039/d0na00556h)
Supplement: NA-002-D0NA00556H-s001 [file NA-002-D0NA00556H-s001.pdf]

Electronic Supplementary Material (ESI) for Nanoscale Advances.
This journal is © The Royal Society of Chemistry 2020

Curie temperature engineering in novel 2D analog of Iron ore (Hematene) via strain

Renu Singla[a], Timothy A. Hackett[b], Sarvesh Kumar[c], Jyotsna Sharma[d] and Manish K. Kashyap[a,*]

[1]Department of Physics, Kurukshetra University, Kurukshetra 136119 (Haryana), India
[b]Department of Biochemistry, University of Nebraska-Lincoln, Lincoln, Nebraska 68588-0664, United States
[c]Inter-University Accelerator Centre (IUAC), Aruna Asaf Ali Marg, New Delhi 110067, India.
[d]Department of Physics, Amity School of Applied Sciences, Amity University Haryana, Gurugram 122413, India
[*]*Corresponding author's e-mail: manishdft@gmail.com, mkumar@kuk.ac.in*

Supplementary Information (SI)

The value of Hubbard U-parameter for pristine hematene was optimized and U = 4 eV was selected because it reproduces the experimental band gap of pristine hematene quite well. It is clearly indicative from Fig. S1 that band gap is underestimated for U = 0, 1.5 and 2.5 eV and overestimated for U = 4.5 eV for both majority and minority spin channels.

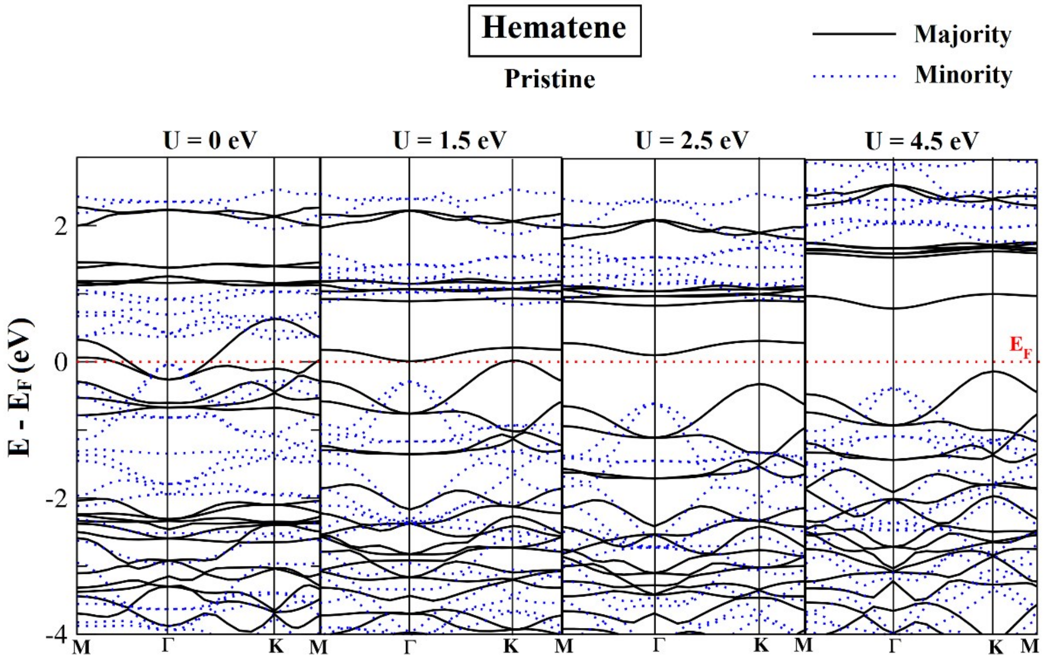

Fig. S1: Calculated spin polarized bandstructures of pristine hematene for different values of Hubbard U-parameter.

The corresponding values of majority/Minority bandgap along Γ-K direction w.r.t. U values are listed in Table S1. For pristine case, the corresponding values are close to

experimental values. As a result of this sensitivity of band gap on U parameter, we have selected U = 4 eV for final calculations of pristine and strain cases.

Table S1: Variation of bandgap (eV) for majority and minority spin channel w.r.t. Hubbard parameter (U).

U (eV)	Bandgap (eV)	
	Majority	Minority
0	-	0.34
1.5	0.00	1.14
2.5	0.41	1.50
4.0	1.00	1.90
4.5	1.10	2.23

Hematene is not flat like graphene and has a thickness of three atoms. Therefore, while simulating the same via DFT approach, the vacuum thickness should be mentioned in between top layer of atoms of first unit cell and the bottom layer of atoms of second unit cell properly. To fulfil this need, a convergence test to select correct vacuum thickness is required, therefore, we performed ground state calculations of hematene by varying vacuum distance from 12 to 20 Å and taking energy cut off as 520 eV. Fig. S2 indicates clearly that ground state energy of hematene is minimum at 15 Å and thus simulation of hematene with vacuum distance of 15 Å can yield the most appropriate results.

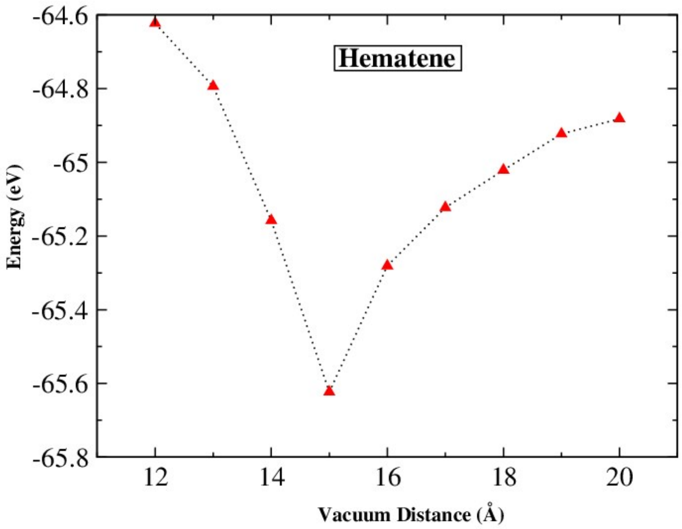

Fig. S2: Convergence test for Vacuum layer thickness for hematene simulations.

Since, the value of MAE is very small with μeV precision, therefore, dependence of its value on energy cut off was also cross checked. For this, we have performed a series of calculations for MAE of pristine hematene by varying energy cut-off from 300 to 600 eV and the corresponding plot is shown in Fig. S3. The plot indicates that there is no change in value of MAE after 450 eV.

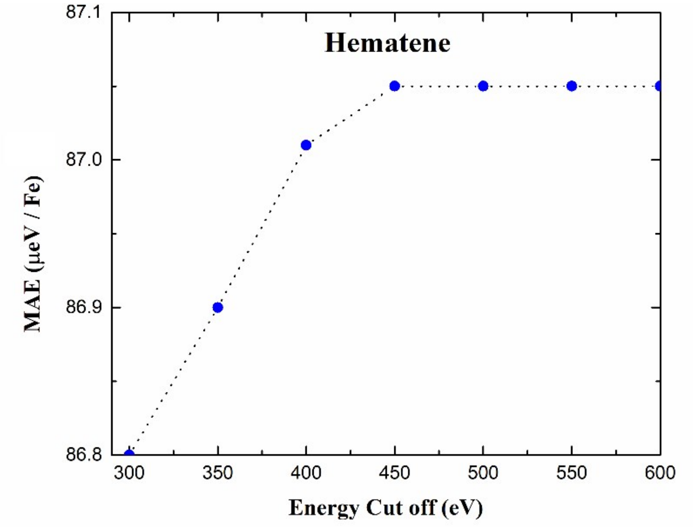

Fig. S3: Variation in MAE (μeV/Fe) of pristine hematene w.r.t. energy cut off (eV).

The formation energy remains negative on increasing both tensile and compressive strain (Fig. S4). This illustrates that with the strain range (-6 % to +6 %), hematene remains structurally stable.

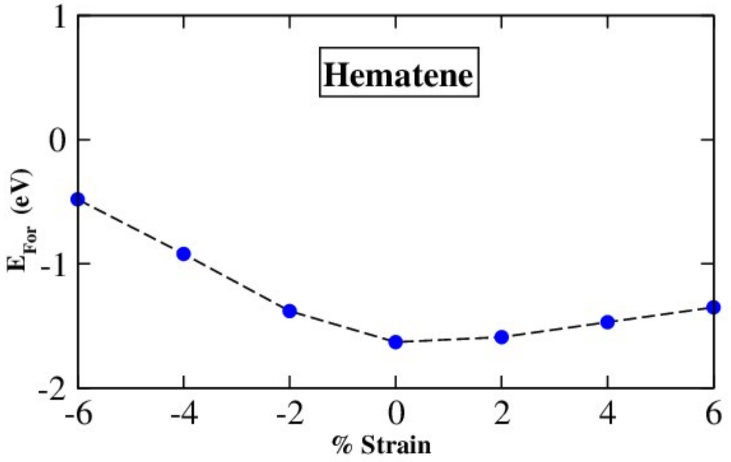

Fig. S4: Formation energy (E_{For}) of pristine and strained hematene.

The stability of strained structures for maximum tensile and compressive strain under consideration (± 6%) was further checked with the help of phonon spectra. The corresponding spectra for ± 6% cases are depicted in Fig. S5. The presence of only positive frequencies indicates the vibrational stability.

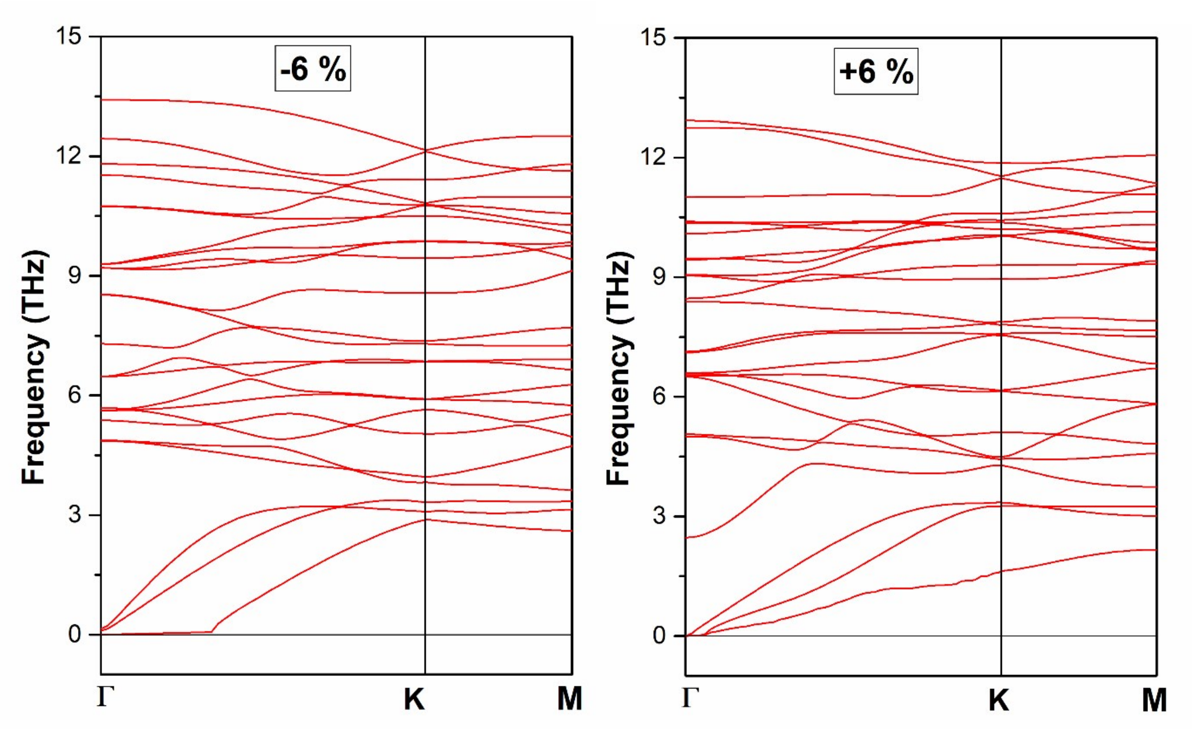

Fig. S5: Phonon dispersion curves for strained hematene (± 6%) along high symmetry k-points.
